# Supplementary material for: Top influencers can be identified universally by combining classical centralities
Source: Sci Rep. 2020 Nov 25;10:20550. doi: 10.1038/s41598-020-77536-7 (PMC7688979; doi:10.1038/s41598-020-77536-7)
Supplement: Supplementary file 1 — Supplementary material 1 [file 41598_2020_77536_MOESM1_ESM.pdf]

Top influencers can be identified universally by combining classical centralities

Doina Bucur[1,*]

[1]University of Twente, Department of Computer Science, Drienerlolaan 5, 7522 NB Enschede, The Netherlands
[*]d.bucur@utwente.nl

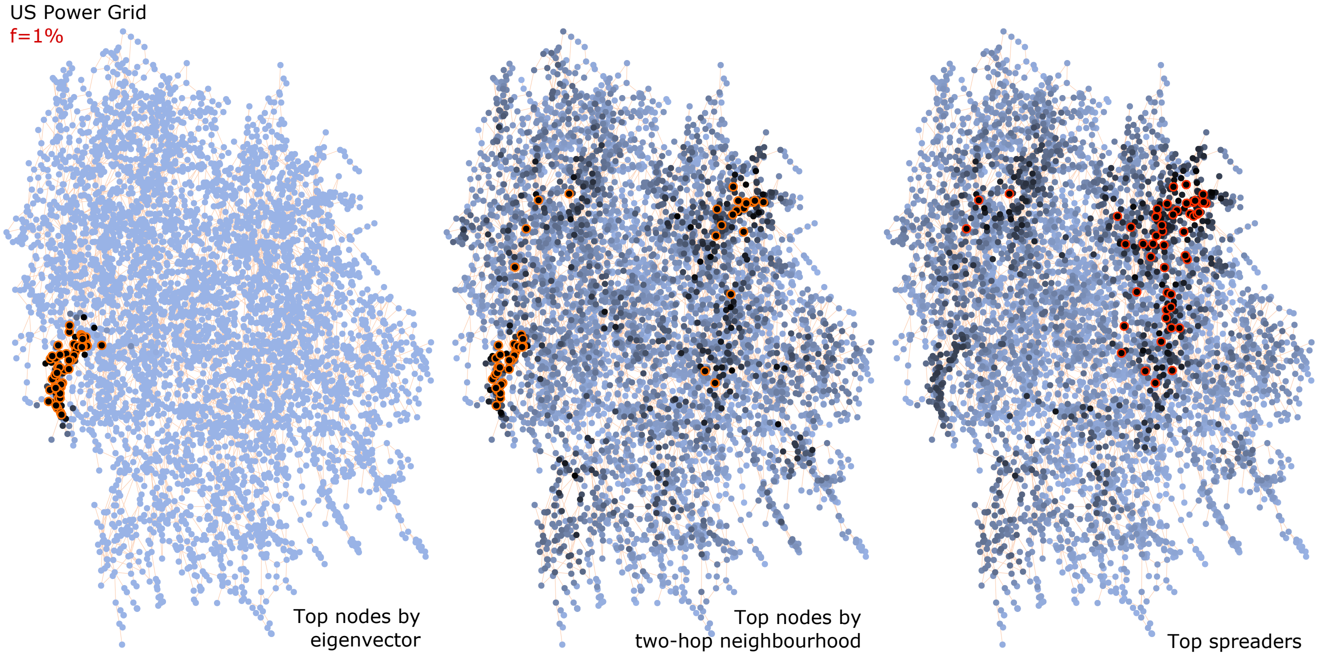

Supplementary Figure S1. Comparing the location of the top nodes as ranked by (left) eigenvector centrality, (centre) two-hop neighbourhood size, and (right) SIR spread size at the epidemic threshold for the infrastructure network US Power Grid. The network layout is force-directed. The colour of the nodes in each panel shows the value of that metric: darker nodes have higher centrality values or spread size. The top $f = 1\%$ of the nodes in each case are encircled.

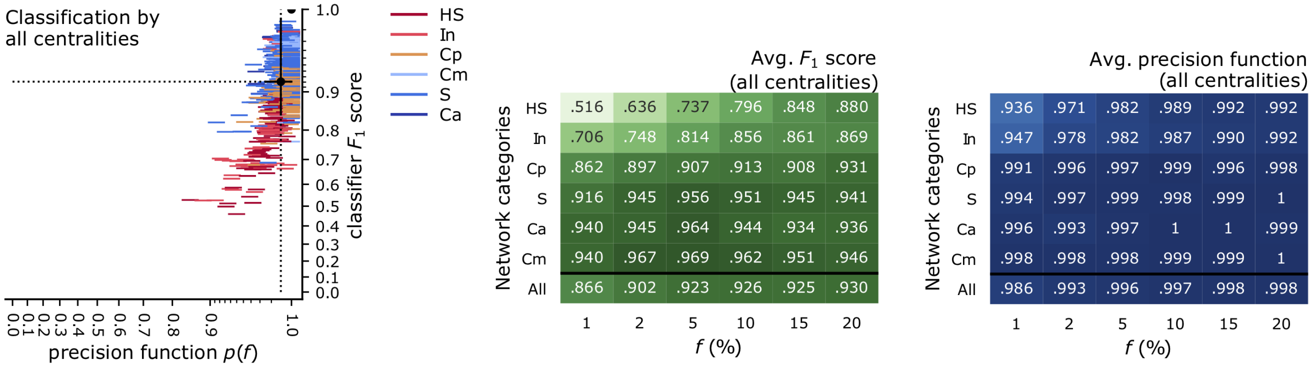

Supplementary Figure S2. The success of **SVM classifiers using all centralities** at predicting spreaders, across all networks and values of f, **above the SIR epidemic threshold**, at $1.5 \cdot \lambda_c$ for each network. (Counterpart to the summary results at the epidemic threshold λ_c from Figure 10).

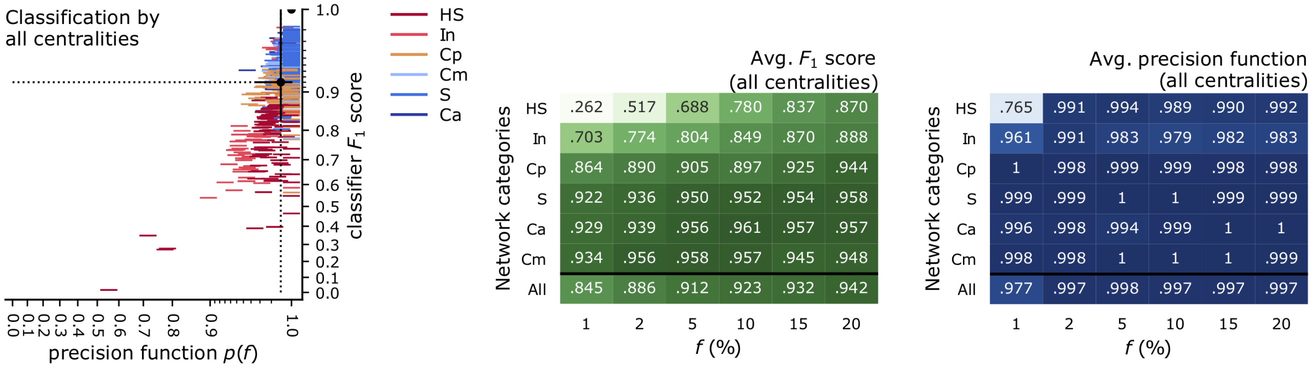

Supplementary Figure S3. The success of **Random Forest classifiers using all centralities** at predicting spreaders, across all networks and values of f, **at the SIR epidemic threshold** λ_c for each network. (Counterpart to the summary results for SVM classifiers at the epidemic threshold λ_c from Figure 10).

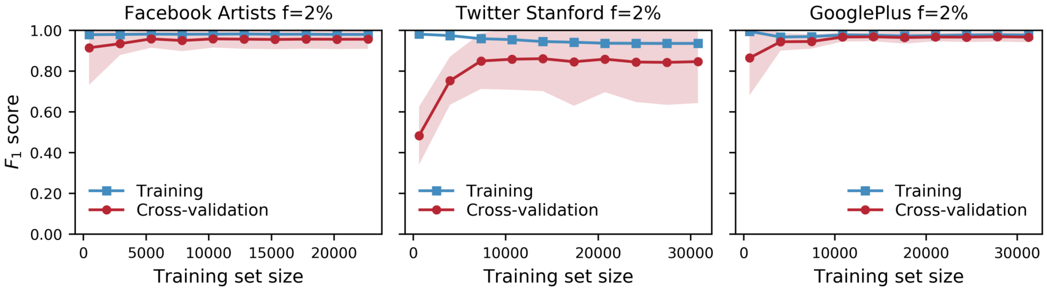

Supplementary Figure S4. Learning curves for three large networks, showing the amount of training data (in terms of the number of nodes, on the x axis) versus the performance after training and cross-validation with all centralities (on the y axis). The training set size ranges between 1% and 50% of the network size, for each network. One can choose the size of the training data; a size is sufficient if the learning curve shows a high cross-validation score, close to the training score.
